# Nutritional status and eating disturbances among patients with neurocognitive disorder

**DOI:** 10.1002/alz70858_105540

**Published:** 2025-12-26

**Authors:** Witsarut Nanthasi, Arthita Choolam, Chatchawan Rattanabannakit, Natthamon Wongkom, Vorapun Senanarong

**Affiliations:** ^1^ Faculty of Medicine Siriraj Hospital, Mahidol University, Bangkoknoi, Bangkok, Thailand; ^2^ Chulabhorn Hospital, HRH Princess Chulabhorn College of Medical Science, Chulabhorn Royal Academy, Bangkoknoi, Bangkok, Thailand; ^3^ Faculty of Medicine Siriraj Hospital, Mahidol University, Bangkok, Thailand

## Abstract

**Background:**

Nutritional status constitutes a significant concern among individuals with cognitive impairment. A strong correlation exists between malnutrition and cognitive function. Nutritional status can be assessed through various methods, including questionnaire, biological indices, and biochemical markers. Individuals experiencing cognitive decline also exhibit swallowing and eating disturbance, which can adversely impact their nutritional status.

**Method:**

Sixty‐two patients diagnosed with cognitive impairment were recruited from the memory clinic. Cognitive impairment severity was categorized based on the Clinical Dementia Rating scale Sum of Boxes (CDR‐SB). Participants underwent a comprehensive assessment including: Mini‐Nutritional Assessment (MNA) questionnaire, Body Mass Index (BMI) and Waist‐Hip Ratio (WHR) measurements, biochemical markers such as total protein, albumin, and hemoglobin levels, and a comprehensive questionnaire to evaluate swallowing and eating disturbances. This questionnaire consisted of five domains: swallowing disturbance, appetite change, food preference, eating habits, and other eating behaviors. The relationship between cognitive impairment severity and nutritional status was analyzed using logistic regression and chi‐square.

**Result:**

Participants were classified into three groups: mild cognitive impairment (MCI) (*n* = 30), mild dementia (*n* = 22), and moderate dementia (*n* = 10). No significant differences were observed between the groups in terms of BMI, WHR, total protein, albumin, and hemoglobin levels. Based on the Mini‐Nutritional Assessment (MNA) score, the odds of malnutrition were found to increase with increasing severity of cognitive impairment. Moderate dementia patients exhibited a significantly higher risk of malnutrition compared to MCI patients (adjusted Odds Ratio [aOR] = 8.9; 95% Confidence Interval [CI] = 0.5 ‐ 3.9; *p* = 0.01). Furthermore, patients with dementia demonstrated a higher proportion of disturbances in eating habits: MCI (36.7%), mild dementia (50.0%), moderate dementia (90.0%); *p* = 0.01. Moderate dementia patients exhibited significantly higher odds of experiencing disturbances in eating habits compared to MCI patients (aOR = 15.5; 95% CI = 1.7 ‐ 139.7; *p* = 0.01).

**Conclusion:**

The severity of cognitive impairment emerged as a significant predictor of both malnutrition risk and the occurrence of eating disturbances.

